# Natural Infection with *Giardia* Is Associated with Altered Community Structure of the Human and Canine Gut Microbiome

**DOI:** 10.1128/mSphere.00670-20

**Published:** 2020-08-05

**Authors:** Alexander S. F. Berry, Kaylynn Johnson, Rene Martins, Megan C. Sullivan, Camila Farias Amorim, Alexandra Putre, Aiysha Scott, Shuai Wang, Brianna Lindsay, Robert N. Baldassano, Thomas J. Nolan, Daniel P. Beiting

**Affiliations:** a Department of Pathobiology, School of Veterinary Medicine, University of Pennsylvania, Philadelphia, Pennsylvania, USA; b Department of Pediatric Gastroenterology, Hepatology and Nutrition, Children’s Hospital of Philadelphia, Philadelphia, Pennsylvania, USA; c Department of Clinical Sciences and Advanced Medicine, School of Veterinary Medicine, University of Pennsylvania, Philadelphia, Pennsylvania, USA; d Department of Biology, School of Arts and Sciences, University of Pennsylvania, Philadelphia, Pennsylvania, USA; University of Utah

**Keywords:** GEMS, *Giardia*, MAL-ED, database, diarrhea, microbiome, parasite

## Abstract

Enteric parasitic infections are among the most prevalent infections in lower- and middle-income countries (LMICs) and have a profound impact on global public health. While the microbiome is increasingly recognized as a key determinant of gut health and human development, the impact of naturally acquired parasite infections on microbial community structure in the gut, and the extent to which parasite-induced changes in the microbiome may contribute to gastrointestinal symptoms, is poorly understood. Enteric parasites are routinely identified in companion animals in the United States, presenting a unique opportunity to leverage this animal model to investigate the impact of naturally acquired parasite infections on the microbiome. Clinical, parasitological, and microbiome profiling of a cohort of 258 dogs revealed a significant correlation between parasite infection and composition of the bacterial community in the gut. Relative to other enteric parasites, *Giardia* was associated with a more pronounced perturbation of the microbiome. To compare our findings to large-scale epidemiological studies of enteric diseases in humans, a database mining approach was employed to integrate clinical and microbiome data. Substantial and consistent alterations to microbiome structure were observed in *Giardia*-infected children. Importantly, infection was associated with a reduction in the relative abundance of potential pathobionts, including *Gammaproteobacteria*, and an increase in *Prevotella*—a profile often associated with gut health. Taken together, these data show that widespread *Giardia* infection in young animals and humans is associated with significant remodeling of the gut microbiome and provide a possible explanation for the high prevalence of asymptomatic *Giardia* infections observed across host species.

**IMPORTANCE** While enteric parasitic infections are among the most important infections in lower- and middle-income countries, their impact on gut microbiota is poorly understood. We reasoned that clinical symptoms associated with these infections may be influenced by alterations of the microbiome that occur during infection. To explore this notion, we took a two-pronged approach. First, we studied a cohort of dogs naturally infected with various enteric parasites and found a strong association between parasite infection and altered gut microbiota composition. *Giardia*, one of the most prevalent parasite infections globally, had a particularly large impact on the microbiome. Second, we took a database-driven strategy to integrate microbiome data with clinical data from large human field studies and found that *Giardia* infection is also associated with marked alteration of the gut microbiome of children, suggesting a possible explanation for why *Giardia* has been reported to be associated with protection from moderate to severe diarrhea.

## Abstract (precis)

While enteric parasitic infections are among the most important infections in lower- and middle-income countries, their impact on gut microbiota is poorly understood. We reasoned that clinical symptoms associated with these infections may be influenced by alterations of the microbiome that occur during infection. To explore this notion, we took a two-pronged approach. First, we studied a cohort of dogs naturally infected with various enteric parasites and found a strong association between parasite infection and altered gut microbiota composition. *Giardia*, one of the most prevalent parasite infections globally, had a particularly large impact on the microbiome. Second, we took a database-driven strategy to integrate microbiome data with clinical data from large human field studies and found that *Giardia* infection is also associated with marked alteration of the gut microbiome of children, suggesting a possible explanation for why *Giardia* has been reported to be associated with protection from moderate to severe diarrhea.

## INTRODUCTION

Enteric parasites, including helminths and protozoa, are among the most prevalent infections in lower- and middle-income countries (LMICs) with an estimated 3.5 billion people affected worldwide (1, 2). Infection with eukaryotic pathogens often results in acute, moderate to severe diarrheal disease and/or chronic malnutrition and stunting, which has significant consequences for morbidity and mortality (3–5). Conversely, some intestinal parasites are frequently associated with asymptomatic infections (6, 7). *Giardia*, for example, was found in 18 of 1,093 (1.6%) of healthy volunteers in Melbourne, Australia (8) and in 286 of 1,359 (21%) of healthy schoolchildren in Madrid, Spain (9). It is important to understand whether and how these abundant and pervasive parasites impact gut health.

While the microbiome is increasingly recognized as a key determinant of gut health and human development, the impact of naturally acquired parasite infections on the microbial community in the gut is poorly understood. Many studies of parasites and their impact on the microbiome involve experimental infections of laboratory animals (10–15). While such studies can be powerful for elucidating mechanisms, they often involve laboratory-adapted parasite strains, specialized animal husbandry practices, or high infectious doses, all of which can impact host immunity and the composition of the microbiome. Conversely, studies of parasite infections in human populations are challenging due to the relatively low prevalence of these infections in developed countries and the presence of confounding variables in LMICs, such as malnourishment and coinfections (16–19). These issues are largely overcome by studying enteric parasite infections in companion animals. Various enteric parasites are frequently found in screenings of domestic dogs and cats in the United States (20). For example, a study of over one million dogs throughout the United States in 2006 found that 12.5% were infected with at least one enteric parasite, with the most prevalent being *Giardia* which infected 4% of dogs (21). As companion animals, dogs are increasingly recognized as an ideal model system for translational gut microbiome research. In addition to harboring similar gut microbiota as humans, dogs often share their environment with humans, consume a similar omnivorous diet, and can spontaneously develop gastrointestinal (GI) disease that shares many features in common with inflammatory bowel disease in humans (22–29). In addition, like humans, dogs frequently become infected with enteric parasites in early life. Here, we performed 16S rRNA sequencing of fecal samples from 258 dogs naturally infected with one or more eukaryotic parasites to evaluate the impact of parasite infection on gut microbiota composition. We found that parasite infections are associated with significant perturbations to the microbiome and that *Giardia* is associated with the largest changes in canine gut microbiota.

We also investigated whether *Giardia*—a frequent infection among humans residing in LMICs—causes similar perturbations in human gut microbiota composition. The Global Enteric Multicenter Study (GEMS) investigated the causes of pediatric moderate to severe diarrhea (MSD) in LMICs (30). In addition to reporting a strong association between infection with rotavirus or *Cryptosporidium* and the development of MSD, this study also reported the surprising observation that *Giardia* was found more often among asymptomatic participants than those with MSD in this cohort (30), despite the association between *Giardia* and serious chronic health conditions, including growth stunting (31), irritable bowel syndrome (IBS), and fatigue (32). A follow-up to the GEMS study performed 16S sequencing of fecal samples from approximately 1,000 GEMS participants (33), but this study only considered the relationship between the microbiome and MSD and did not examine a role for parasite infections in influencing this relationship. Similarly, The Etiology, Risk Factors, and Interactions of Enteric Infections and Malnutrition and the Consequences for Child Health (MAL-ED) study investigated the hypothesis that infection with enteric pathogens contributes to undernutrition in children (34). A follow-up to the MAL-ED study performed 16S sequencing on nearly 1,000 fecal samples from participants in the Peruvian cohort to assess the taxa associated with the burden of *Campylobacter* and other enteric pathogens (35). We used a database mining approach to determine whether *Giardia* infection perturbs the human gut microbiome, in both the GEMS and MAL-ED cohorts, similarly to how it perturbs the canine gut microbiome, and to gain insight into possible mechanisms by which *Giardia* infection may be linked to protection against diarrhea in some individuals.

## RESULTS

### Enteric parasite infections perturb the canine microbiome.

**FIG 1:**
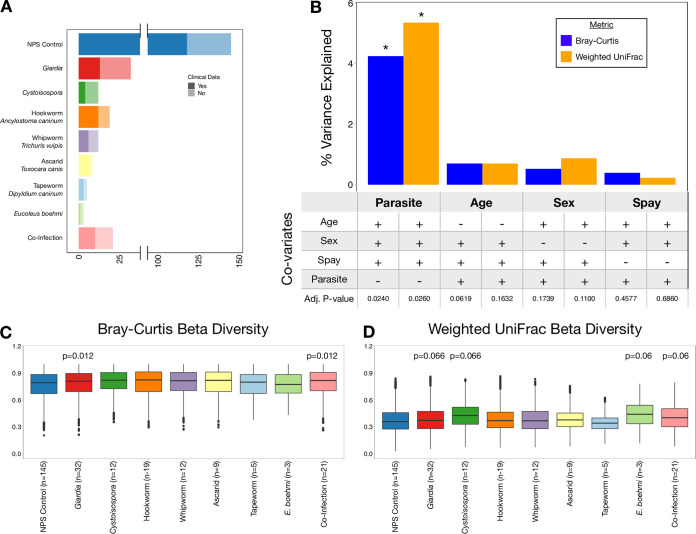
Parasite infection perturbs canine gut microbiota. 16S sequencing of fecal samples from 258 dogs infected with none, one, or multiple enteric parasites was performed. (A) The number of no parasite seen (NPS) controls, dogs infected with each parasite, and dogs infected with more than one parasite are shown. The number of dogs for which clinical data are unavailable are represented by lighter shading. (B) The percent variance in Bray-Curtis and weighted UniFrac beta diversity explained by each variable is represented by blue and orange bars, respectively. Whether or not age, sex, spay/neuter status, or parasite infection were controlled and the significance of each variable are shown below each bar. Asterisks highlight variables with adjusted *P* values of <0.05. (C and D) Boxplots showing the difference in Bray-Curtis beta diversity (C) and weighted UniFrac beta diversity (D) between samples in each infection and NPS controls. Adjusted *P* values of <0.1 are shown above each box.

A stool bank was generated from samples screened at a veterinary clinical parasitology service as part of our Companion Animal Microbiome during Parasitism (CAMP) study (see Materials and Methods). A total of 258 canine fecal samples were split into 9 groups based on parasite infection status (Fig. 1A): (i) no parasite seen (NPS) controls; (ii) *Giardia*, the causative agent of giardiasis; (iii) *Cystoisospora*, the causative agent of coccidiosis, an intestinal tract infection; (iv) hookworm, which causes intestinal distress and anemia; (v) whipworm, which causes severe irritation to the large intestine; (vi) ascarid, which causes weakness, diarrhea, and vomiting; (vii) tapeworm, which is generally asymptomatic but indicative of flea infestation and may cause perianal pruritis; (viii) Eucoleus boehmi, a parasite whose eggs are shed in the stool and that can cause chronic rhinitis; and (ix) dogs with coinfections of two or more of these parasites. Since certain enteric parasites, such as *Giardia*, are more prevalent in young animals, age and other potential confounding variables were controlled for in our statistical analyses. Parasite infection status was associated with significant changes in beta diversity, as determined by both Bray-Curtis and weighted UniFrac metrics, even when covariates such as age, sex, and spay/neuter status were controlled for as confounding variables (adjusted *P* value [Adj *P*] < 0.05 by permutational multivariate analysis of variance [PERMANOVA]) (Fig. 1B). The significance of parasite infection remains unchanged when infections represented by fewer than 9 samples were removed from the analysis (tapeworm and *Eucoleus boehmi*). Approximately 5% of the variation in microbiome composition was explained by parasite infection status compared to <1% explained by age, sex, or spay/neuter status alone (Adj *P* > 0.05 by PERMANOVA) (Fig. 1B). Specifically, *Giardia-* and coinfected animals displayed the most significant differences in beta diversity compared to NPS controls by both Bray-Curtis (Fig. 1C) and weighted UniFrac metrics (Fig. 1D).

### Canine *Giardia* infection is associated with significant alterations in gut microbiota composition.

**FIG 2:**
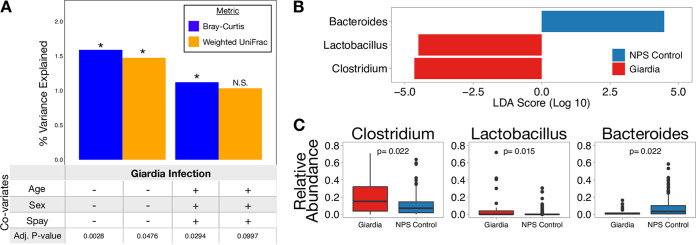
*Giardia* infection is associated with enrichment of several key bacterial taxa in the canine gut. (A) Histogram showing that *Giardia* infection is associated with a significant difference in beta diversity compared to NPS controls. Bar height reflects the percentage of total beta diversity variance that is explained by *Giardia* infection. Plus symbols below the bars show when age, sex, and spay/neuter status are controlled for. Asterisks denote bars with adjusted *P* values of <0.05. (B) LEfSe graph shows the magnitude of enrichment with LDA score of > comparing *Giardia*-infected dogs to NPS control dogs. (C) Boxplots showing the relative abundance of differentially enriched taxa. *Clostridium* is among the most highly enriched bacterial taxa associated with *Giardia* infection compared to controls.

Given the diverse range of parasites detected in our animals, we set out to determine whether specific types of parasites were associated with more pronounced microbiome alterations. *Giardia* infection is associated with a change in Bray-Curtis (Adj *P* < 0.01; 1.6% of total variation) and weighted UniFrac (Adj *P* < 0.05; 1.5% of total variation) beta diversity compared to NPS controls, without controlling for age, sex, and spay/neuter status (Fig. 2A). When controlling for age, sex, and spay/neuter status, beta diversity is still significantly altered during *Giardia* infection as measured by Bray-Curtis (Adj *P* < 0.05; 1.1% of total variation), but no longer meets the 0.05 cutoff for significance for weighted UniFrac (Adj *P* = 0.0997; 1.0% of total variation) (Fig. 2A). The differences in beta diversity between *Giardia* infection and NPS controls were driven by several bacterial taxa as determined by linear discriminant analysis (LDA) effect size (LEfSe) analysis (Fig. 2B and C). *Giardia* is associated with enrichment of *Clostridium*, a genus that contains several commensal taxa, as well as an enrichment of *Lactobacillus*. However, *Giardia* was also associated with a reduction in *Bacteroides*, a genus that includes important commensal bacteria. In order to verify the taxa associated with *Giardia* infection, point-biserial correlation coefficients were calculated for each taxon with average relative abundance of >1%. Consistent with our LEfSe results, point-biserial correlation coefficients also showed enrichment of *Clostridium* and *Lactobacillus*, and a reduction in *Bacteroides* in addition to a reduction in *Megamonas* (see Table S1 in the supplemental material). The high relative abundance of *Clostridium* and *Lactobacillus* and the low relative abundance of *Bacteroides* in *Giardia*-infected dogs compared with NPS controls show that *Giardia* infection in animals is associated with an altered gut microbiota composition.

10.1128/mSphere.00670-20.7 TABLE S1 Differentially abundant taxa associated with *Giardia* infection compared to NPS controls, as identified by point-biserial correlation coefficient. Point-biserial correlation coefficients showing that *Clostridium*, *Lactobacillus*, and *Bacteroides* are significantly associated with *Giardia* infection, as seen in the LEfSe analysis (Fig. 2). Download Table S1, PDF file, 0.04 MB. Copyright © 2020 Berry et al. 2020 Berry et al. This content is distributed under the terms of the Creative Commons Attribution 4.0 International license.

It is possible that the microbiota changes observed in *Giardia-*infected dogs could be driven, in part, by clinical variables such as diarrhea or antibiotic use. To discriminate between changes linked to diarrhea or antibiotics versus those linked to infection, we evaluated medical records, when available (*n* = 174), to identify animals with a recent history of diarrhea or antibiotic use (Fig. 1A). Interestingly, *Giardia* infection was not associated with diarrhea or antibiotic use (*P* > 0.25 by chi-squared test): among dogs with clinical data, 4 of 13 (30.1%) *Giardia*-infected dogs had diarrhea compared to 21 of 118 (18%) NPS control dogs with diarrhea (see Fig. S1A in the supplemental material), while 4 of 13 (15.4%) *Giardia*-infected dogs received antibiotics compared to 15 of 118 (12.7%) NPS control dogs (Fig. S2A). In contrast, antibiotic use was strongly correlated with diarrhea (*P* < 0.01; chi-squared test), with most dogs on antibiotics having diarrhea (11/15) and over half of dogs with diarrhea being on antibiotics (11/21). Among NPS control animals with clinical data available (*n* = 118 dogs) (Fig. S1A), those with diarrhea had significantly different Bray-Curtis beta diversity (Adj *P* < 0.001;, 2.9% of total variation) and weighted UniFrac beta diversity (Adj *P* < 0.01; 3.7% of total variation) compared to asymptomatic animals; and those receiving antibiotics had significantly different Bray-Curtis beta diversity (Adj *P* < 0.001, 3.5% of total variation) and weighted UniFrac beta diversity (Adj *P* < 0.01; 3.9% of total variation) compared to those not receiving antibiotics, when controlling for age, sex, and spay/neuter status (Fig. S1B). Next, we used our NPS control group (*n* = 118) to define a microbiome signature associated with diarrhea in the absence of observable parasites, allowing us to compare this signature with *Giardia*-infected animals. LEfSe analysis identified *Escherichia* as enriched in animals with diarrhea and in those receiving antibiotics, while *Bacteroides* and *Fusobacterium* were enriched in asymptomatic dogs (Fig. S1C and S1D) and those not receiving antibiotics (Fig. S2B). Taken together, these data define a microbiome profile associated with diarrhea and antibiotic use in NPS animals that is marked by enrichment of *Escherichia* and *Fusobacterium* and show that this signature is distinct from that observed during *Giardia* infection (Fig. 2B and C).

10.1128/mSphere.00670-20.1 FIG S1 Diarrhea is associated with enrichment of a different set of taxa compared to *Giardia*. (A) Pie charts showing the relative proportion of dogs with diarrhea and asymptomatic animals among NPS control and *Giardia*-infected dogs. The proportion of symptomatic dogs is not significantly different between groups (*P > *0.05 by chi-squared test). (B) Histogram showing that diarrhea and antibiotic use are associated with a significant difference in beta diversity compared to asymptomatic and no antibiotic use. Bar height reflects the percentage of total beta diversity variance that is explained by each variable. Age, sex, and spay/neuter status were controlled for all calculations. Asterisks denote bars with adjusted *P* < 0.05. (C) LEfSe graph showing the magnitude of enrichment for each taxon with a LDA score of >2 comparing animals with and without diarrhea. (D) Boxplots showing the relative abundance of taxa that are differentially abundant between animals with and without diarrhea. Download FIG S1, PDF file, 0.1 MB. Copyright © 2020 Berry et al. 2020 Berry et al. This content is distributed under the terms of the Creative Commons Attribution 4.0 International license.

10.1128/mSphere.00670-20.2 FIG S2 Antibiotic use in dogs is associated with a similar gut microbiota profile as dogs with diarrhea due to strong correlation between antibiotic use and diarrhea. (A) Pie chart showing the relative proportion of dogs receiving antibiotics and those not among all samples that have associated clinical data (*n* = 174), NPS controls with clinical data (*n* = 118), and *Giardia*-positive dogs with clinical data (*n* = 15). (B) LEfSe graph showing the magnitude of enrichment for each taxon with LDA score > 2 comparing NPS control dogs receiving and not receiving antibiotics. *Escherichia* is highly enriched in dogs receiving antibiotics and in dogs with diarrhea, while *Bacteroides* and *Fusobacterium* are reduced in dogs receiving antibiotics and in dogs with diarrhea, likely because most dogs receiving antibiotics have diarrhea. *Megamonas* and *Faecalibacterium* are reduced in dogs receiving antibiotics, but not in dogs with diarrhea. Download FIG S2, PDF file, 0.07 MB. Copyright © 2020 Berry et al. 2020 Berry et al. This content is distributed under the terms of the Creative Commons Attribution 4.0 International license.

### The effect of *Giardia* on the microbiome persists during coinfection.

We reasoned that if *Giardia*—compared to other parasites observed in our samples—is driving changes in the microbiome, then we should observe a similar profile in animals harboring coinfections with *Giardia* and at least one other parasite. Ten out of 21 dogs harboring multiple parasites (“coinfection”) were infected with *Giardia* and one or more other parasites. These 10 *Giardia* coinfected samples were indistinguishable from *Giardia* singly infected animals by Bray-Curtis (Adj *P* > 0.1) and weighted UniFrac (Adj *P* > 0.1) beta diversity (Fig. S3). In contrast, *Giardia* singly infected samples were significantly different from the remaining 11 coinfected samples not involving *Giardia* by Bray-Curtis (Adj *P* < 0.05) and weighted UniFrac (Adj *P* < 0.05) beta diversity; however, false discovery rate correction raises these *P* values slightly above the 0.05 significance threshold (Fig. S3). Taken together, these results show that *Giardia* infection in dogs is associated with a unique and significant change in gut microbiota composition compared to NPS controls that persist even in the context of coinfection with other parasites.

10.1128/mSphere.00670-20.3 FIG S3 The effects of *Giardia* in canines persist even when one or more other parasites are present. Fecal samples from dogs infected with multiple parasites, one of which is *Giardia*, are not different from those singly infected with *Giardia* in terms of Bray-Curtis or weighted UniFrac beta diversity (Adj *P > *0.1). In contrast, fecal samples from dogs infected with multiple, non-*Giardia* parasites are different from those singly infected with *Giardia* (*P* < 0.05; adjusted *P* < 0.1), and infection status here represents a larger percentage of the variance in beta diversity. Age, sex, and spay/neuter status were controlled for all calculations. Download FIG S3, PDF file, 0.05 MB. Copyright © 2020 Berry et al. 2020 Berry et al. This content is distributed under the terms of the Creative Commons Attribution 4.0 International license.

### *Giardia* infection is among the largest predictors of the pediatric gut microbiota structure in the GEMS case-control study.

**FIG 3:**
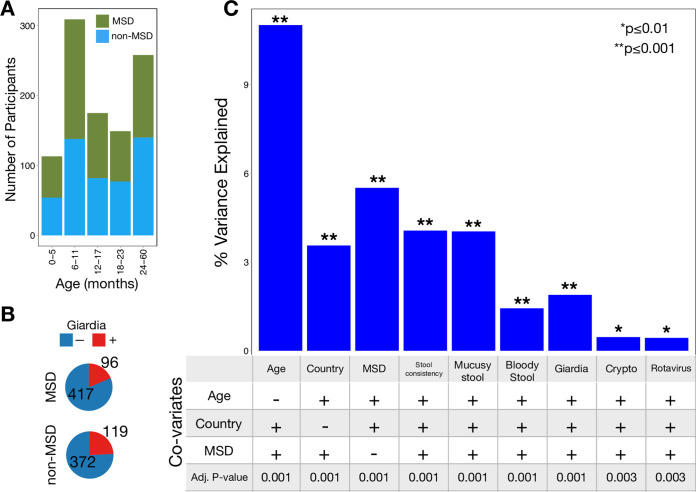
Enteric parasites are associated with gut microbiota perturbations in children. (A) The number of participants with moderate to severe diarrhea (MSD) (green) and without MSD (blue) in each of five age cohorts is shown. (B) *Giardia* is more frequently found in children without MSD compared to children with MSD. (C) The percent variation in Bray-Curtis beta diversity explained by several variables is shown by bars. Whether the analysis was stratified by age, country, and/or MSD status is shown below each bar. *Giardia* is significantly associated with a change in gut microbiota and explains more microbiota variation than any other enteric pathogen detected here.

After finding that parasites, in particular *Giardia*, perturb the canine gut microbiome, we asked whether *Giardia* similarly affected the human gut microbiome. To this end, we employed a database mining approach to integrate and query data from the Global Enteric Multicenter Study (GEMS) and the Etiology, Risk Factors, and Interactions of Enteric Infections and Malnutrition and the Consequences for Child Health (MAL-ED) study. Clinical and epidemiological data from GEMS were made available on ClinEpiDB.org (Fig. 3A and B) from over 22,000 participants. Previously published microbiome data from a subset of the same participants (*n* = 1,004) were loaded on MicrobiomeDB.org (36, 37). Clinical and microbiome data were manually integrated, leading to the identification of 215 participants who were positive for *Giardia* and for which microbiome data were available. Not surprisingly, age and moderate to severe diarrhea (MSD) were strongly correlated with Bray-Curtis beta diversity (Adj *P* < 0.001), explaining 11% and 5.5% of the total variation in microbiome structure, respectively (Fig. 3C). *Giardia* infection was associated with a similarly large perturbation of the gut microbiota (Adj *P* < 0.001; 1.9% of the total variation), while *Cryptosporidium* and rotavirus infection were each associated with <0.5% of the variation in microbiota composition in this cohort (Adj *P* < 0.01). Only 14 of the 215 *Giardia*-infected children were also coinfected with either *Cryptosporidum* or rotavirus. Infection with *Giardia* was also significantly associated with gut microbiota composition in each of the four countries individually (Fig. S4A).

10.1128/mSphere.00670-20.4 FIG S4 *Giardia* infection is associated with significant changes in gut microbiome composition in each of the four countries sampled. (A) Bars depict the percent variation in Bray-Curtis beta diversity explained by *Giardia* infection in each of the four countries. Each analysis was stratified by age and MSD status. *Giardia* is significantly associated with a change in gut microbiota in all four countries; however, the amount of variation explained by *Giardia* infection varies across countries. (B) LEfSe graph showing the magnitude of enrichment for all taxa meeting the adjusted *P* value threshold of 0.1, comparing children with and without *Giardia* infection. Only taxa with relative abundance of >1% across all samples were used in the analysis. Asterisks denote the significance threshold for each taxon in each country. Download FIG S4, PDF file, 0.09 MB. Copyright © 2020 Berry et al. 2020 Berry et al. This content is distributed under the terms of the Creative Commons Attribution 4.0 International license.

**FIG 4:**
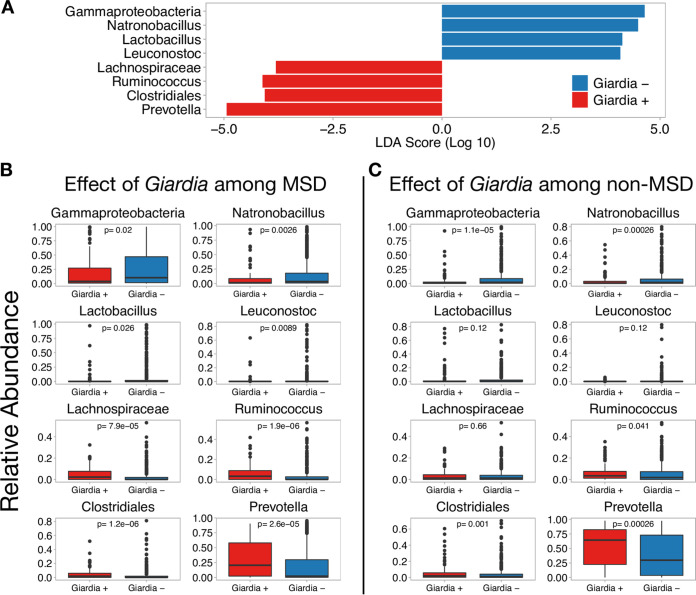
*Giardia* infection in children is associated with a reduction in *Gammaproteobacteria* regardless of disease status. (A) LEfSe graph showing the magnitude of enrichment for each taxon with LDA score of >2 comparing children with and without *Giardia* infection. (B and C) Boxplots showing the relative abundance of differentially enriched taxa among children with MSD (B) and those without MSD (C). A very similar set of taxa are differentially expressed during *Giardia* infection regardless of clinical disease. Although the relative abundance of *Gammaproteobacteria* and *Prevotella* are different between MSD and non-MSD, *Giardia* infection is significantly associated with a reduction of *Gammaproteobacteria* and enrichment of *Prevotella* regardless of MSD status. Taxa were collapsed to the genus level when possible; however, *Gammaproteobacteria*, *Clostridiales*, and *Lachnospiraceae* were only able to be collapsed to the class, order, and family, respectively.

We observed that *Giardia* infection among GEMS participants was associated with enrichment of *Prevotella* and a reduction in *Gammaproteobacteria* (Fig. 4A)—an effect that was evident in children with MSD (Fig. 4B) and without MSD (Fig. 4C). LEfSe analyses performed on data partitioned by country showed that *Giardia* infection is associated with enrichment of *Prevotella* and a reduction in *Gammaproteobacteria* in all four countries (Fig. S4B). Diarrhea is commonly associated with a reduction in *Prevotella* and an increased abundance of *Gammaproteobacteria*. Moreover, age strongly influences the relative abundance of *Prevotella* and *Gammaproteobacteria* (Fig. S5A and S5B, respectively), as well as *Giardia* prevalence (Fig. S5C and S5D). To control for these factors, the impact of *Giardia* was assessed among 12- to 17-month-old GEMS participants, a cohort with high relative abundance of both *Prevotella* and *Gammaproteobacteria* and high prevalence of *Giardia* infections (29.1%; *n* = 51) and for which *Giardia* prevalence is not correlated with age (*P* = 0.99 by chi-squared test). Among 12- to 17-month-old children, the association between *Giardia* infection and reduction in *Gammaproteobacteria* and enrichment of *Prevotella* remained (Fig. S6).

10.1128/mSphere.00670-20.5 FIG S5 *Prevotella* abundance, *Gammaproteobacteria* abundance, and *Giardia* prevalence are correlated with age in young children. (A and B) Boxplots showing that, over the first 5 years of life, the relative abundance of the *Prevotella* genus increases with age (A) and the relative abundance of the *Gammaproteobacteria* class decreases with age (B). (C and D) Bar graphs showing that the proportion of children infected with *Giardia* is low for children in the first year of life compared to 1- to 5-year-old children. Download FIG S5, PDF file, 0.1 MB. Copyright © 2020 Berry et al. 2020 Berry et al. This content is distributed under the terms of the Creative Commons Attribution 4.0 International license.

10.1128/mSphere.00670-20.6 FIG S6 *Giardia* is associated with a reduction in *Gammaproteobacteria* and enrichment of *Prevotella* among 12- to 17-month-old children. (A) LEfSe graph showing the magnitude of enrichment for each taxon with LDA score > 2 comparing 12- to 17-month-old children with (*n* = 51) and without (*n* = 124) *Giardia* infection. (B) Boxplots showing the differences in relative abundance in taxa associated with *Giardia* infection among all 12- to 17-month-old participants. (C and D) Boxplots showing that *Giardia* is associated with a reduction in the *Gammaproteobacteria* class among 12- to 17-month-old children with MSD (C) but that *Giardia* is not significantly associated with *Gammaproteobacteria* among 12 to 17-month-old children without MSD (D), when the relative abundance of *Gammaproteobacteria* is low. Download FIG S6, PDF file, 0.1 MB. Copyright © 2020 Berry et al. 2020 Berry et al. This content is distributed under the terms of the Creative Commons Attribution 4.0 International license.

### Longitudinal tracking of *Giardia* status reveals infection-dependent alterations in microbiome composition.

**FIG 5:**
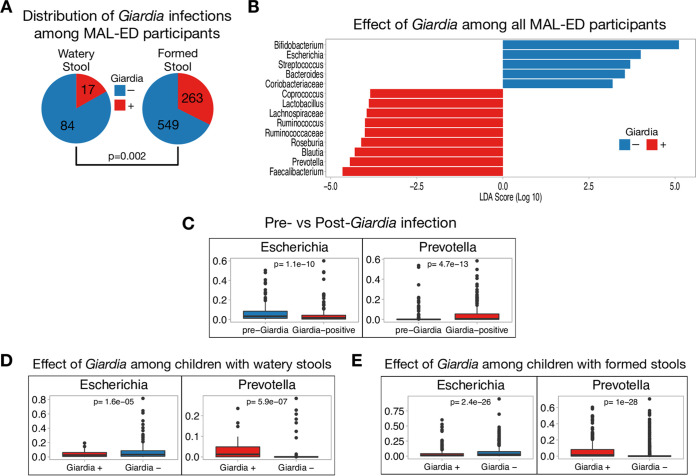
*Giardia* is associated with a reduction in *Escherichia* and enrichment of *Prevotella* among Peruvian children in the MAL-ED study. (A) Among all Peruvian participants in the MAL-ED study, *Giardia* is more frequently found in children with formed or soft stools than in children with liquid or watery stools (*P* = 0.002 by chi-squared test). (B) LEfSe graph showing the magnitude of enrichment for all taxa meeting the adjusted *P* value threshold of 0.05, comparing children with (red) and without (blue) *Giardia* infection. Only taxa with relative abundance of >1% across all samples were used in the analysis. (C) Boxplots showing the differences in relative abundance of *Escherichia* and *Prevotella* immediately before and after acquiring *Giardia* infection. (D) Boxplots showing that *Giardia* is associated with a reduction in *Escherichia* and enrichment of *Prevotella* among children with liquid or watery stools and (E) among children with formed or soft stools. All *P* values were adjusted using Benjamini-Hochberg multiple testing correction.

We employed the same database mining strategy to combine clinical and microbiome data from the MAL-ED study. Clinical and epidemiological data for 2,145 participants sampled longitudinally (>1.8 million observations) were stored on ClinEpiDB.org and were manually integrated with fecal microbiome data (available on microbiomeDB.org) from 271 participants from the Peru cohort, each sampled up to four times at 6, 12, 18, and 24 months of life (*n* = 913 samples) (34, 38). Of the 913 fecal samples, 280 were positive for *Giardia* by enzyme-linked immunosorbent assay (ELISA). *Giardia* was observed more frequently in formed or soft stool samples compared to liquid or watery stools (*P* = 0.002 by chi-squared test) (Fig. 5A), consistent with the GEMS study, where *Giardia* infection was more prevalent among participants without MSD. *Giardia* infection among MAL-ED participants was associated with enrichment of *Prevotella* and a reduction in *Escherichia*, a member of the *Gammaproteobacteria* class (Fig. 5B). MAL-ED sequenced the V4 region of the 16S gene and was classified against the SILVA database, while GEMS sequenced the V1-V2 region and was classified against the Greengenes database, which likely led to some differences in taxonomic classification, including the identification of the *Escherichia* genus specifically in MAL-ED compared to the broader *Gammaproteobacteria* class in GEMS.

We reasoned that if *Giardia* infection was driving the change in microbiome composition observed in the GEMS data, then the longitudinal aspect of MAL-ED should allow identification of individuals that convert from *Giardia* negative to positive during the study period, providing a unique context for examining parasite-microbiome interactions. A total of 170 pairs of MAL-ED observations were identified where a *Giardia*-positive stool sample could be compared to the *Giardia*-negative sample obtained from the same individual 6 months prior. Analysis of the microbiome in these discordant pairs showed that becoming infected with *Giardia* was marked by decreased relative abundance of *Escherichia* and increased *Prevotella* (Adj *P* < 1e−9) (Fig. 5C). Since samples were collected 6 months apart, an effect of age cannot be ruled out. To address this, we also evaluated 53 pairs of observations where individuals converted from Giardia positive to negative over a 6-month period. In these cases, clearing *Giardia* was not associated with a change in relative abundance of *Escherichia* or *Prevotella* (Adj *P* > 0.1), suggesting that the differences observed upon infection cannot be explained by age alone. These data also suggest that clearing *Giardia* does not have an inversely proportional effect on the gut microbiota composition as acquiring *Giardia*. Finally, the enrichment of *Prevotella* and reduction in *Escherichia* were evident in children with liquid or watery stools (Fig. 5D) as well as those with formed or soft stools (Fig. 5E). Taken together, these results demonstrate that *Giardia* infection leads to an altered gut microbiome structure in humans, marked by changes in the relative abundance of taxa linked to gut health (39–41).

## DISCUSSION

Enteric parasite infections are among the most common causes of diarrhea in humans in the developing world. While bacterial infections and the gut microbiome have been well-studied, the impact of enteric eukaryotic parasites on the microbiome is not well understood, with some reports showing altered microbiome composition (17, 42–46, 105) while others showed either modest or no impact (47–50). Because these studies often rely on experimental infection with one or few parasite species or observations using a small number of participants, they provide limited insight into the broader impact of enteric parasites on the gut microbiome. By combining clinical parasitology and microbiome profiling from humans and canines infected with a phylogenetically diverse range of enteric parasites, we show that naturally acquired enteric parasite infections are a major factor associated with microbiome composition, that this effect is observed across host species, and that *Giardia* is associated with the largest impact among all parasites surveyed in dogs and humans.

*Giardia* is one of the most common enteric parasites in the world and is remarkable in its ability to cause an array of clinical phenotypes, ranging from asymptomatic infection to severe acute diarrheal disease to chronic gastrointestinal disease. *Giardia* is the causative agent of giardiasis, a diarrheal illness, and is clearly implicated in serious growth stunting and long-term health consequences (51), cementing its role as a pathogen. However, our observations (Fig. 3), as well as other reports in humans and animals suggest that *Giardia* infection is frequently asymptomatic (8, 51–58). Intriguingly, several large epidemiological case-control studies recently showed higher *Giardia* prevalence in asymptomatic participants compared to those with moderate to severe diarrheal disease, revealing a possible protective role (30, 34, 51, 56, 59, 60). The negative association between *Giardia* and MSD may be due, in part, to asymptomatic participants shedding hardier cysts that preserve DNA better than the trophozoites typically associated with severe *Giardia* infection; however, data from this study and others suggest biological explanations for the phenomenon. *Giardia* infection may modulate symptoms in some individuals by modulating the immune response to other pathogens (56, 61), as seen in a recent study showing that *Giardia* coinfection attenuates the severity of disease caused by other enteric pathogens (62). We reasoned that parasite-induced perturbations in the microbiome could also be an important factor influencing gastrointestinal symptoms. Our results, in combination with similar results from recent smaller-scale mouse and human experiments (62, 63), raise the possibility that the shift in microbiome composition during *Giardia* infection—marked by a reduction in *Gammaproteobacteria* and an increase in *Prevotella*—may explain, at least in part, the apparent protective effect of *Giardia* against diarrhea in some age/site cohorts (30, 34, 56).

One intriguing extension of our findings is the notion that *Giardia* may benefit directly from manipulation of the microbiome as seen during infection with the intestinal parasite *Blastocystis* which engulfs highly abundant bacterial taxa to meet its nutritional demands, causing drastic changes to gut microbiota composition (64). Infection by another protozoan parasite, Entamoeba histolytica, results in enrichment of Escherichia coli that protects the parasite from oxidative damage by producing malate dehydrogenase (65). Similarly, during infection with the helminth Trichuris muris, *Proteobacteria* directly interact with parasite eggs to induce hatching, thereby enhancing worm reproduction (66). Taken together, these studies highlight that eukaryotic parasites impact the microbiome in ways that can influence host health, immunity, and parasite biology.

Our data show an association between *Giardia* infection and microbiome composition but do not resolve whether infection is the primary driver of these changes. It is possible that certain microbiome compositions confer susceptibility or resistance to colonization by the parasite, as suggested by previous studies (67). Although it may seem surprising that a pathogen of the upper small intestine could have the potential to impact microbiome composition in the stool, recent studies of mice experimentally infected with *Giardia* revealed alterations of the gut microbiome throughout the small and large intestine, indicating both a causal role for infection in inducing these changes and the ability of the parasite to profoundly alter bacterial community structure far from the site of infection (12). Moreover, our analysis of longitudinal data from the MAL-ED study, which show a clear shift in composition commensurate with infection, argue in favor of microbiome changes being a consequence, rather than a cause, of infection.

Taken together, these data make a strong case for pursuing mechanistic studies that address interactions between *Giardia*, the microbiome, and the host. Interestingly, *Giardia* infection is associated with malabsorption of fats, leading to intestinal steatosis and increased transit of lipids into the distal small intestine and colon (68), malabsorption of sugars and proteins, and diffuse shortening of the intestinal brush border microvilli (69, 70). These pathological changes could alter substrate availability for commensal bacteria, providing a possible explanation for compositional changes in the microbiome during this infection. Additionally, gut microbiota composition changes could be caused by *Giardia*-induced disturbances to biofilm composition and structure which have been linked to dysbiosis (71, 72).

The age at which humans or animals are exposed to *Giardia* is thought to impact clinical manifestations. For example, there appears to be a window of time early in childhood development when *Giardia* infection is negatively associated with diarrhea. Studies of several pediatric cohorts show either no correlation or a negative correlation between *Giardia* infection and diarrhea (30, 51, 53–55, 73), although definitions of diarrhea vary from “loose stool” to clinically severe diarrhea. Notably, those studies that specify moderate to severe diarrhea show a significant association between *Giardia* infection and not having diarrhea (30, 54), suggesting that *Giardia* infection may be negatively associated with severe diarrhea but not loose stools. Indeed, clinical manifestations associated with *Giardia* infection are variable, likely due in part to variation among *Giardia* genotypes, and may explain why children in countries where *Giardia* is not endemic are more likely than children in countries where *Giardia* is endemic to have symptomatic *Giardia* infection (74–77). In contrast, adults—especially those in areas where *Giardia* is not endemic—show a positive correlation between *Giardia* infection and diarrhea (51, 78). Previous studies suggest that the association between growth stunting and *Giardia* infection is dependent on age (79), with some studies showing that asymptomatic *Giardia* infection is associated with growth stunting among children older than 18 months, but not infants or in children during their first 18 months (80), while others show an association between *Giardia* and growth stunting at 2 years of age (31). The effects of *Giardia* on gut microbiota may also be age dependent. The MAL-ED study of Peruvian children found that gut microbiota associated with *Giardia* burden varied by age (35). For example, high *Giardia* burden was associated with enrichment of *Prevotella* only in fecal samples of 24-month-old children. Here, we show an association between *Giardia* infection and altered gut microbiota composition in specific age cohorts as well, raising the possibility that parasite-microbiome interactions may partially explain the age-dependent disease presentation during *Giardia* infection. Collectively, these data point to the gut microbiome, host immunity, parasite genotype, and age (81–84) as variables that may interact or operate independently to augment the balance between protection and pathogenesis during *Giardia* infection.

One major obstacle to investigating relationships between clinical variables and microbiome composition in large-scale studies like GEMS and MAL-ED is that these data are not always collected at the same time, by the same researchers, with the goal of being analyzed together. For example, although extensive clinical and epidemiological data were collected from over 22,000 participants in GEMS (30) and over 1.8 million observations in MAL-ED (34), microbiome profiling data were collected from a subset of approximately 1,000 samples from each study, and were published separately and with relatively sparse metadata (33, 38). Our study highlights that a database-driven approach that integrates microbiome data with extensive clinical and epidemiological data allows for the identification of novel associations and an opportunity to compare microbiome phenotypes across host species and across studies.

## MATERIALS AND METHODS

A dockerized environment containing code and software is available on Code Ocean (https://codeocean.com/capsule/2815529/tree) and fully reproduces all analyses and figures in the article.

### Canine sample collection.

Fecal samples for our Companion Animal Microbiome during Parasitism (CAMP) study were acquired from patients seen at the Ryan Hospital at the University of Pennsylvania’s School of Veterinary Medicine (PennVet) as part of both sick and wellness visits, as well as from healthy dogs in animal shelters that were brought to the Ryan Hospital to be spayed or neutered. Fecal samples were examined for parasites by fecal flotation (using a zinc sulfate solution at a specific gravity of 1.18 g/ml) at the Clinical Parasitology Laboratory of PennVet. Dogs either had no observable parasites (*n* = 145), one (*n* = 92), or multiple (*n* = 21) protozoan parasites, including *Giardia* (*n* = 32) and *Cystoisospora* (*n* = 12), and helminths, including hookworm (Ancylostoma caninum) (*n* = 19), whipworm (Trichuris vulpis) (*n* = 12), ascarid (Toxocara canis) (*n* = 9), tapeworm (Dipylidium caninum) (*n* = 5), and Eucoleus boehmi (*n* = 3) (Fig. 1A). Although zinc flotation is less sensitive for detecting *Giardia* infection compared to antigen immunoassays, it has the advantage of higher specificity for detecting active *Giardia* infections rather than detecting antigen that can persist even after the infection is cleared. Samples containing yeast were excluded from the study. Age, sex, and spay and neuter status were recorded at the time of fecal sample collection for all samples. Fecal samples from 113 infected dogs and 145 dogs without detectable parasites were stored at −80°C until DNA extraction. Clinical data from patient visits were obtained for 174 PennVet patients to determine whether gastrointestinal symptoms or antibiotic use occurred within 1 week of fecal sample collection.

### 16S rRNA gene sequencing and analysis.

DNA was extracted from fecal samples using Qiagen PowerSoil DNA extraction kit. 16S rRNA gene sequencing was performed as described previously (85). Briefly, the V4 region of the 16S rRNA gene was amplified using PCR, which was performed using Accuprime Pfx supermix and custom primers for 30 cycles (85). PicoGreen quantification was used to normalize post-PCR products, and AMPureXP beads were used to clean the combined pools. Libraries were quantified and sized using a Qubit 2.0 and Tapestation 4200, respectively. Then, 250-bp paired-end sequencing was performed using an Illumina MiSeq. The QIIME2 pipeline (86) was used to process and analyze 16S sequencing data. Samples were demultiplexed using q2-demux and denoised using Dada2 (87). Sequences were aligned using maaft (88), and phylogenetic trees were reconstructed using fasttree (89). Weighted UniFrac (90) and Bray-Curtis (91) beta diversity metrics were estimated using q2-core-metrics-diversity after samples were rarefied to 4,100 reads per sample, and *P* values were adjusted for multiple hypothesis testing using Benjamini-Hochberg (B-H) false discovery rate (FDR) corrections (92). Taxonomy was assigned to sequences using q2-feature-classifier classify-sklearn (93) against the Greengenes 13-8 99% operational taxonomic unit (OTU) reference sequences (94). Taxa were collapsed to the genus level, when possible. OTUs with less than 1% average relative abundance across all samples were removed.

### Correlation analysis and differential feature selection.

The correlation between variables such as parasite infection and microbiota composition was determined using permutational multivariate analysis of variance (PERMANOVA) as implemented in the vegan package (95) in R (96). Differentially abundant taxa were determined using linear discriminant analysis (LDA) effect size (LEfSe) (97), and *P* values were adjusted for multiple hypothesis testing using B-H FDR corrections in R. Boxplots and LEfSe plots were visualized using ggplot2 (98), patchwork (99), and ggthemes (100). Point-biserial correlation coefficients were calculated to identify differentially abundant taxa between *Giardia*-infected and no parasite seen (NPS) controls with 10,000 permutations using the indicspecies package in R (101), adjusting for multiple hypothesis testing using B-H FDR corrections (see Table S1 in the supplemental material).

### Integration and analysis of GEMS data.

The Global Enteric Multicenter Study (GEMS) investigated the causes, incidence, and impact of moderate to severe diarrhea in 23,567 0- to 59-month-old children in Asia and Africa (30). Clinical and epidemiological data and anthropometric measurements for each participant were downloaded from ClinEpiDB.org (36, 37). The presence of *Giardia*, *Cryptosporidium*, and rotavirus were determined using ELISA on participant fecal samples. Additionally, sequencing of the V1-V2 region of the 16S rRNA gene was performed on stool samples from 1,007 participants (33). Taxonomy was determined by classifying sequences against the Greengenes 99% OTU reference sequences. Here, clinical data from 1,004 GEMS participants was downloaded from ClinEpiDB.org, and the relative abundances of bacterial taxa for the same 1,004 participants was downloaded from MicrobiomeDB.org (102). The data sets were manually combined so that clinical and epidemiological data were matched to gut bacterial taxon abundance data.

Correlations between clinical variables (e.g., *Giardia* infection) and Bray-Curtis beta diversity were calculated using the vegan package in R. Patients were divided among five age groups (0 to 6, 6 to 12, 12 to 18, 18 to 24, and 24 to 59 months) to control for the effects associated with age. Here, associations with age were stratified by country, associations with country were stratified by age, and all other associations were stratified by age group and country (Fig. 5C), as done by Kotloff et al. (30). Taxonomy was collapsed to the genus level, when possible, and taxa with mean relative abundance across all samples of <1% were removed. Differentially abundant taxa between *Giardia*-positive versus *Giardia*-negative and MSD cases versus controls were determined using LEfSe, adjusting *P* values for multiple hypothesis testing using B-H FDR corrections. LEfSe plots and boxplots were visualized using ggplot2 (98), patchwork (99), and ggthemes (100).

### Integration and analysis of MAL-ED data.

The Etiology, Risk Factors, and Interactions of Enteric Infections and Malnutrition and the Consequences for Child Health (MAL-ED) study investigated the burden of enteropathogens and malnutrition by monitoring 2,145 children across eight sites in Africa, Asia, and South America starting at 17 days of life and followed longitudinally for up to 60 months (34). Clinical and epidemiological data and anthropometric measurements for each participant were downloaded from ClinEpiDB.org (36, 37). Fecal samples were collected from participants, and the presence of *Giardia* was determined by ELISA. Additionally, sequencing of the V4 region of the 16S rRNA gene was performed on stool samples from 913 participants from the Peruvian cohort (38). Taxonomy was determined by classifying sequences against the SILVA reference database (103, 104). Here, clinical data from 913 MAL-ED observations were downloaded from ClinEpiDB.org, and the relative abundances of bacterial taxa for the same observations were downloaded from MicrobiomeDB.org (102). The data sets were manually combined so that clinical and epidemiological data were matched to gut bacterial taxa abundance data. Taxonomy was collapsed to the genus level, when possible, and taxa with mean relative abundance across all samples of <1% were removed. Differentially abundant taxa between *Giardia*-positive versus *Giardia*-negative children were determined using LEfSe, adjusting *P* values for multiple hypothesis testing using B-H FDR corrections. The effects of *Giardia* on gut microbiota composition were also stratified by stool consistency: the effects among watery or liquid stools and the effects among formed or soft stools. LEfSe plots and boxplots were visualized using ggplot2 (98), patchwork (99), and ggthemes (100).

### Data availability.

All sequencing data analyzed here are publicly available on the Sequence Read Archive (SRA) under study accession number PRJNA594732. All sequencing data used for the canine analyses is also publicly available on MIcrobiomeDB.org as part of the CAMP study. All code is available and reproducible on CodeOcean (https://codeocean.com/capsule/2815529/tree).
