# Supplementary material for: Natural Infection with Giardia Is Associated with Altered Community Structure of the Human and Canine Gut Microbiome
Source: mSphere. 2020 Aug 5;5(4):e00670-20. doi: 10.1128/mSphere.00670-20 (PMC7407069; doi:10.1128/mSphere.00670-20)
Supplement: TABLE S1 [file mSphere.00670-20-st001.pdf]

| Taxa enriched in Giardia-infected compared to NPS Controls | | |
|---|---|---|
| **Taxa** | **Correlation Coefficient** | **Adj. P-value** |
| *Clostridium* | 0.29 | 0.014 |
| *Lactobacillus* | 0.23 | 0.014 |

| Taxa enriched in NPS Controls compared to Giardia-infected | | |
|---|---|---|
| **Taxa** | **Correlation Coefficient** | **Adj. P-value** |
| *Bacteroides* | 0.31 | 0.013 |
| *Megamonas* | 0.28 | 0.018 |
