# Supplementary figures and images for: Natural Infection with Giardia Is Associated with Altered Community Structure of the Human and Canine Gut Microbiome
Source: mSphere. 2020 Aug 5;5(4):e00670-20. doi: 10.1128/mSphere.00670-20 (PMC7407069; doi:10.1128/mSphere.00670-20)

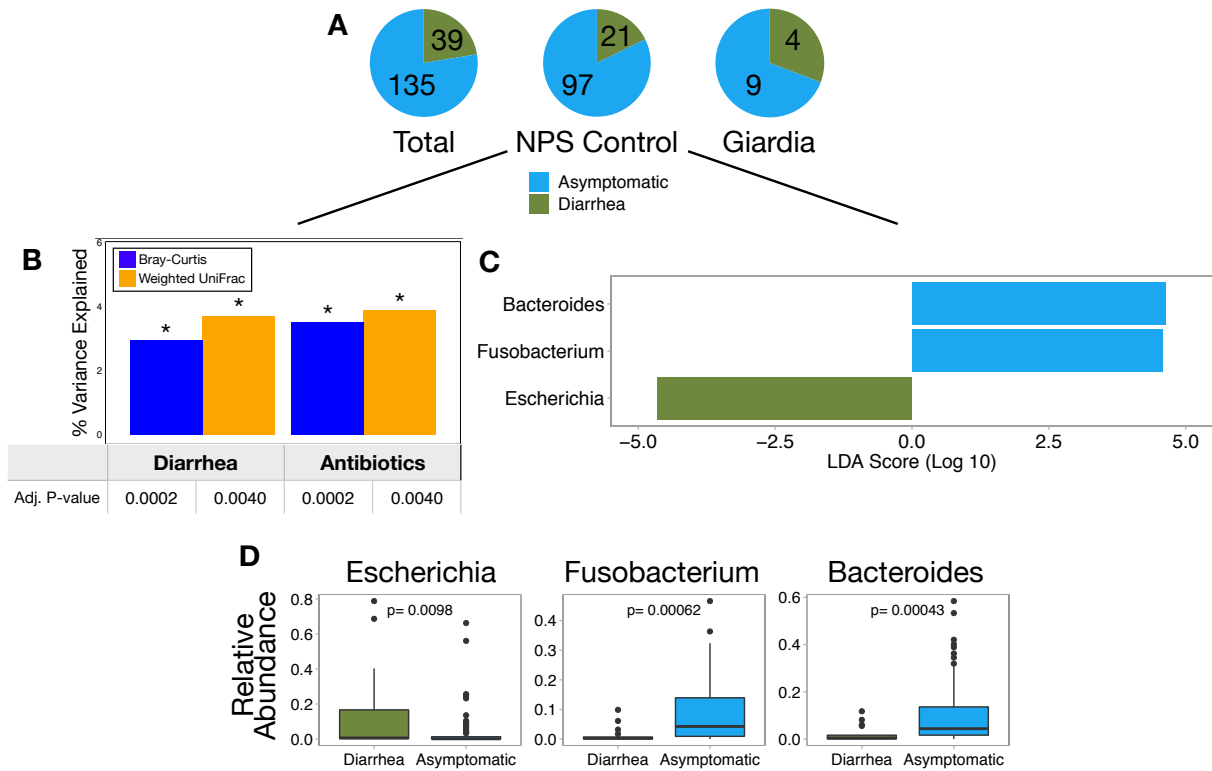

Supplement: FIG S1 [file mSphere.00670-20-sf001.pdf]

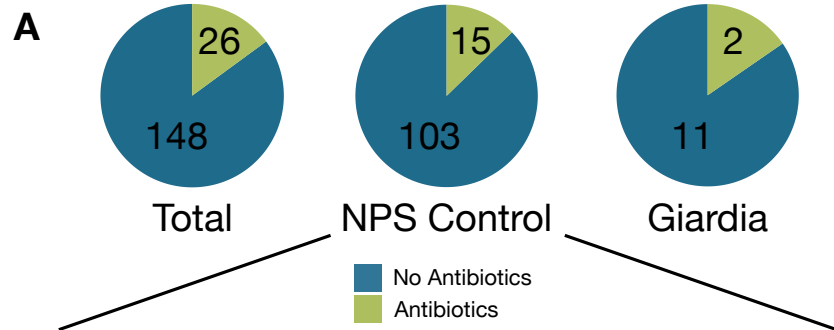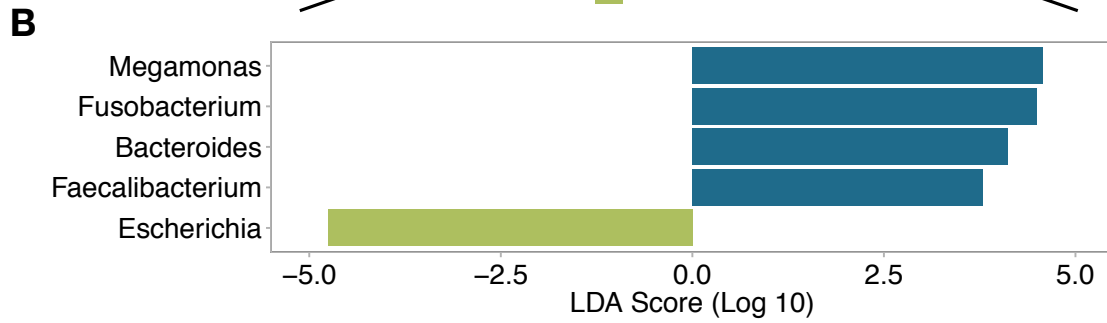

Supplement: FIG S2 [file mSphere.00670-20-sf002.pdf]

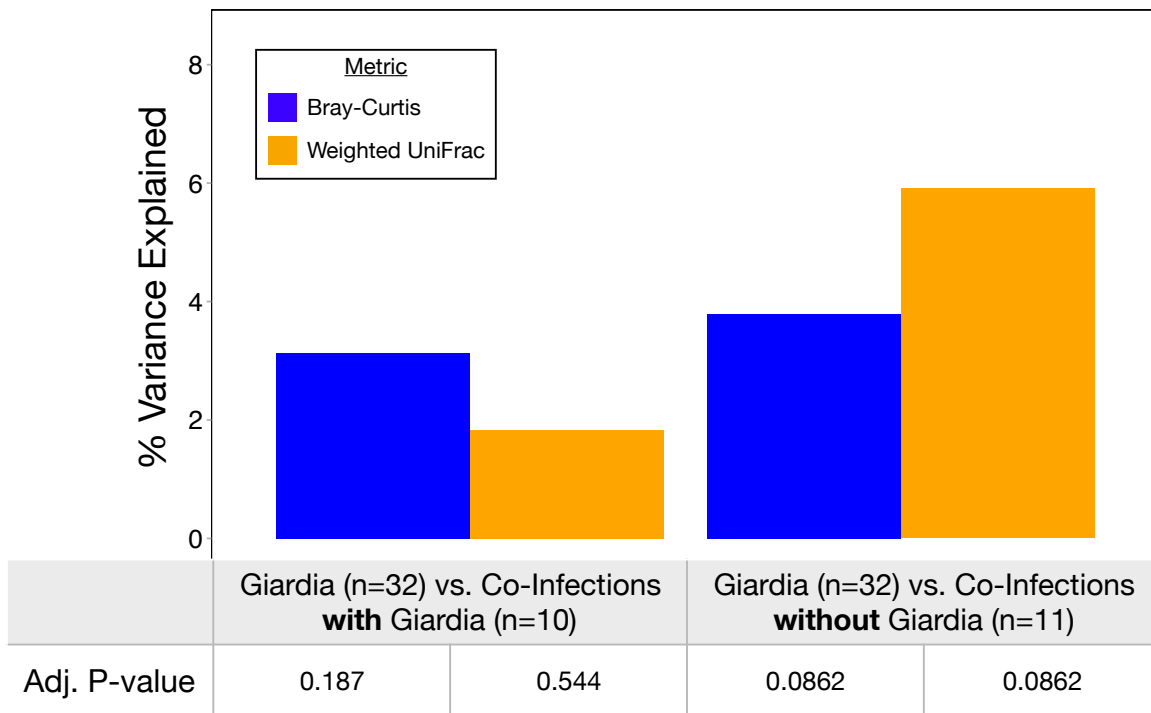

Supplement: FIG S3 [file mSphere.00670-20-sf003.pdf]

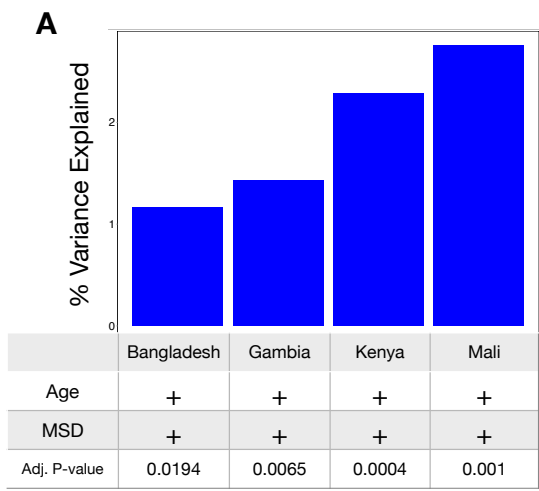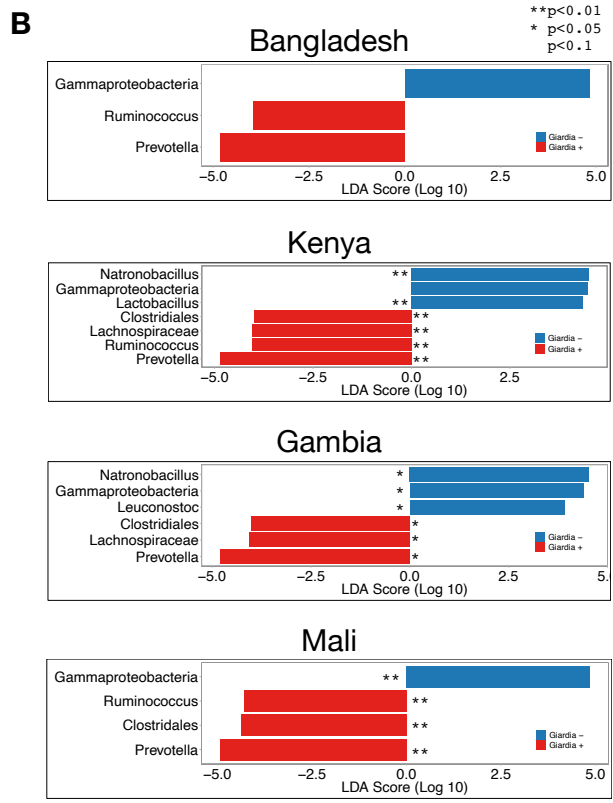

Supplement: FIG S4 [file mSphere.00670-20-sf004.pdf]

**A****Prevotella**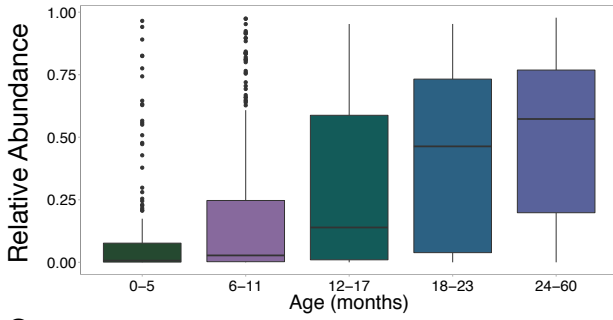**B****Gammaproteobacteria**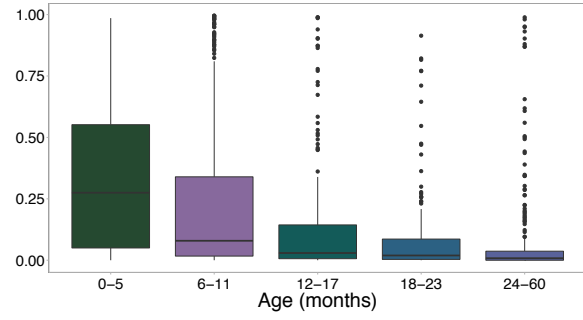**C****Giardia Prevalence**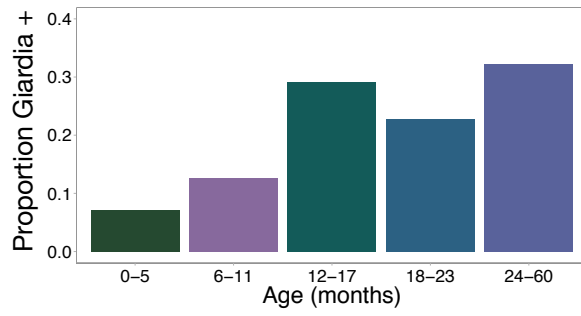**D****Giardia Prevalence**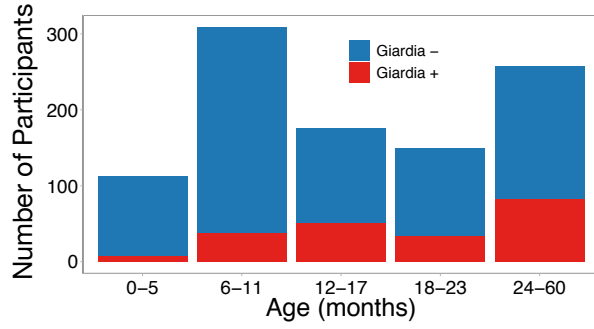

Supplement: FIG S5 [file mSphere.00670-20-sf005.pdf]

**A**

Among all 12-17 month-old children

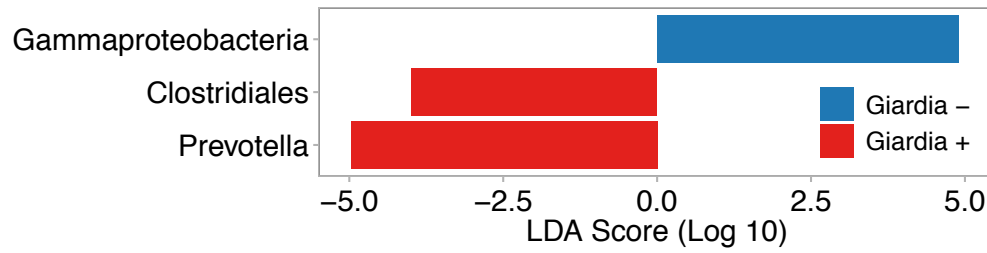**B**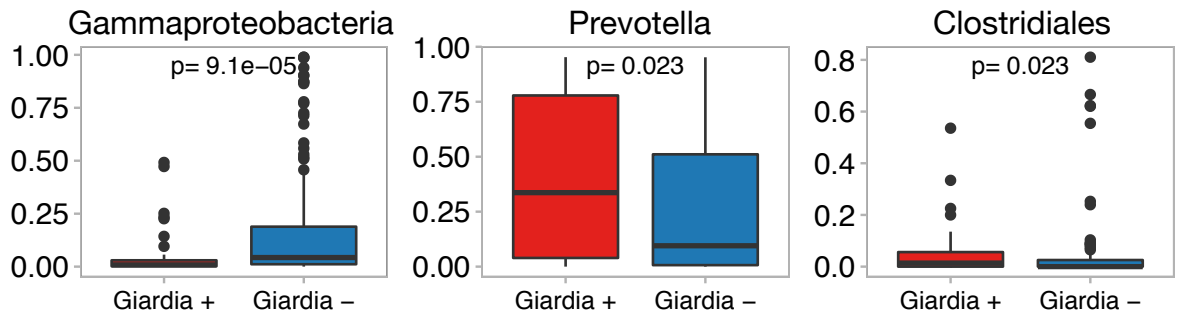**C**

12-17 MSD

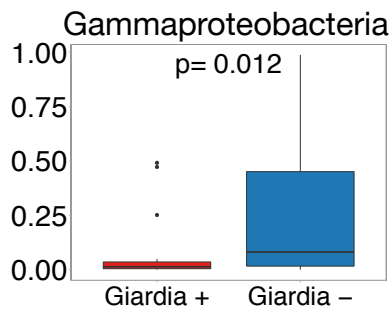**D**

12-17 non-MSD

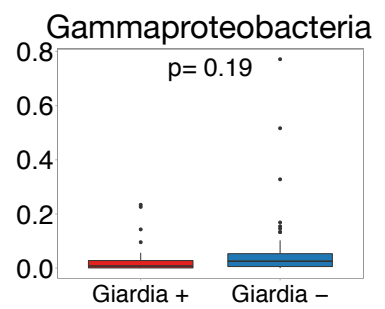

Supplement: FIG S6 [file mSphere.00670-20-sf006.pdf]
